# Creation and Evaluation of a Cesarean Section Simulator Training Program for Novice Obstetric Surgeons

**DOI:** 10.7759/cureus.10324

**Published:** 2020-09-09

**Authors:** Lisa M Foglia, Allison A Eubanks, Logan C Peterson, Kimberly Hickey, Crystal B Hammons, Lindsey B Borgia, Morgan R Light, Amanda Jackson, Shad Deering

**Affiliations:** 1 Maternal Fetal Medicine, Womack Army Medical Center, Fort Bragg, USA; 2 Obstetrics and Gynecology, Walter Reed National Military Medical Center, Bethesda, USA; 3 Maternal Fetal Medicine, Walter Reed National Military Medical Center, Bethesda, USA; 4 Obstetrics and Gynecology, Madigan Army Medical Center, Tacoma, USA; 5 Obstetrics and Gynecology, Fort Belvoir Community Hospital, Fort Belvoir, USA; 6 Obstetrics and Gynecology, Christus Health, San Antonio, USA

**Keywords:** simulation in medical education, simulation trainer, emergency and elective cesarean, postpartum hemorrhage, education and training of medical students and doctors (specialist and phd)), deployment medicine

## Abstract

Background: We evaluated a novel simulation-based cesarean section training program to teach critical techniques for cesarean section and hemorrhage management.

Methods: This was a prospective educational intervention. After Institutional Review Board approval, we recruited Obstetrics and Gynecology, Family Medicine, and General Surgery residents at three hospitals. All participants received didactic education. Participants were then randomized into two arms with one group to receive task-trainer based training and the other no training. Afterwards, all residents had their performance of a complete cesarean section and management of a post-partum hemorrhage evaluated on a high-fidelity simulator. Evaluators were blinded to randomization.

Experience: Thirty-three participants were recruited between July 2017 and January 2019. There were 19 trainees in the control group and 14 in the intervention group. The intervention group scored significantly higher on performance of the cesarean delivery (p-value 0.007), hemorrhage management (p-value 0.0002), and overall skill (p-value 0.008). There were no differences in the other categories.

Conclusion: Participants trained with a combination of didactic education and task-trainers versus didactic education alone performed significantly better on all procedural aspects of a cesarean section and hemorrhage management on a high-fidelity simulator, demonstrating that simulation-based training allows trainees to gain procedural experience while decreasing patient risk.

## Introduction

Simulation has the potential to increase surgical skills and prepare physicians to perform complex tasks without risk to patients. Training using simulators has increasingly been incorporated into all aspects of obstetrics education. The Accreditation Council for Graduate Medical Education (ACGME) now requires simulation to be utilized in Obstetrics and Gynecology (OB/GYN) training programs [1]. In 2018, the American Board of Obstetrics and Gynecology made completion of the Fundamentals of Laparoscopic Surgery (FLS) program a prerequisite for the certifying examination [2,3]. Obstetric simulation demonstrates positive effects on clinical outcomes for shoulder dystocia, operative vaginal delivery, emergent cesarean delivery, postpartum hemorrhage, and can improve teamwork in response to obstetric emergencies [2-9]. Non-obstetricians may benefit from simulation training in the event they need to perform or assist in a cesarean section during a combat deployment, while on a humanitarian mission, or when stationed at a remote facility [10,11]. Simulation may also be useful in rural or low resource areas where an experienced OB/GYN may not be immediately available. As such, a high-fidelity cesarean section surgical simulator may have benefit when used as an adjunct to learn, practice, and reinforce obstetric surgical skills in a wide variety of settings.

Our objective was to evaluate the performance of a high-fidelity simulator program to train novice providers on cesarean delivery and post-partum hemorrhage management.

## Materials and methods

This study was a prospective educational intervention approved by the Institutional Review Boards from all institutions involved, conducted from July 2017 to January 2019. Residents from three separate military training hospitals were invited to participate by a study administrator who had no role in residency oversight or evaluation. The administrator obtained informed consent and allocated trainees to didactic versus didactic plus task trainer. We offered enrollment to all OB/GYN interns, Family Medicine senior residents, and General Surgery senior residents. Trainees were excluded if they previously served as primary surgeon for a cesarean section during their residency. This was a convenience sample, based on the total number of trainees available for participation.

**Figure 1:**
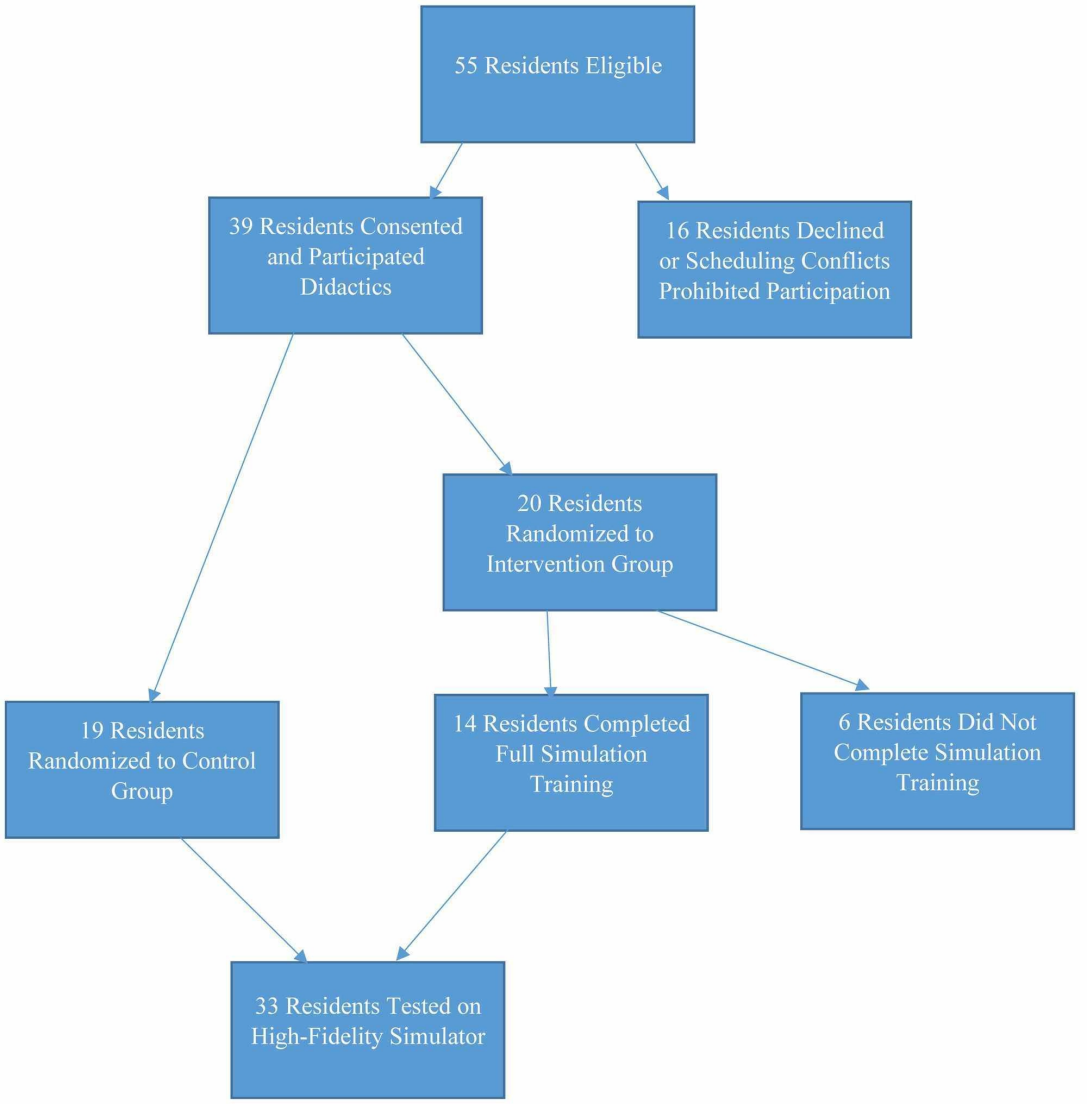
Flow Chart of Paricipant Inclusion

All participants attended a standardized didactic lecture covering the indications for cesarean, procedural steps of the case, and medical and surgical postpartum hemorrhage management techniques. After the lecture, trainees were informed about whether they were allocated to the didactic education only versus didactic education plus task-trainer group. The task-trainer group underwent training with cesarean section task-specific simulators for the procedures of skin incision, uterine incision, delivery of the fetus, wound closure, and operative interventions for post-partum hemorrhage including placement of uterine compression sutures and repair of a uterine artery laceration. The didactic-only group did not receive any simulation training. After the lecture and simulation training, an evaluation was scheduled approximately six to eight weeks later (Figure 1).

**Figure 2:**
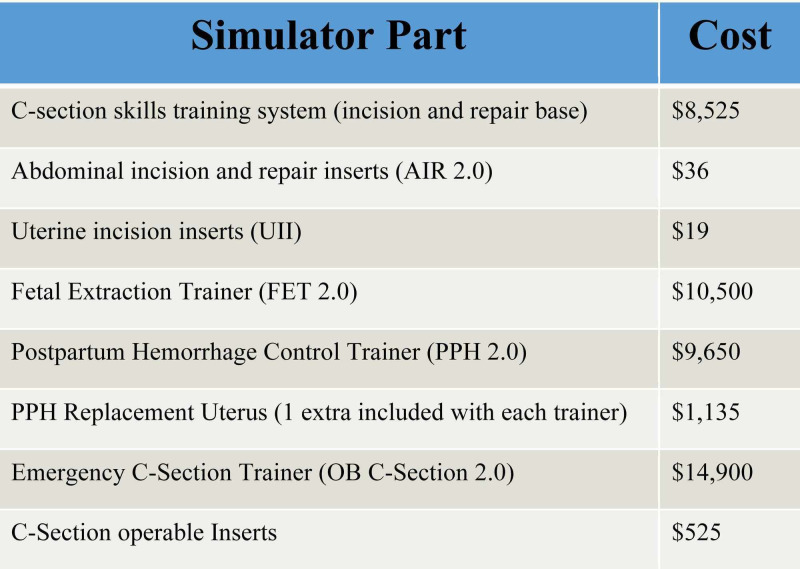
Simulator Cost Breakdown

**Figure 3:**
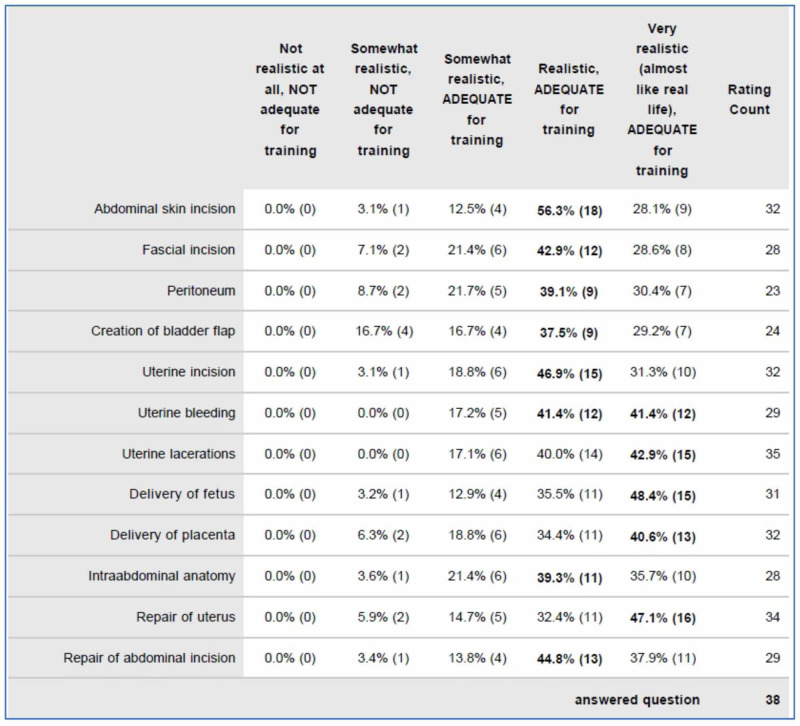
Initial Review of Simulator

A high-fidelity trainer made by Operative Experience^©^ was used for the skills evaluation (Figure 2). Thirty-eight experienced obstetricians had previously assessed this simulator and 94.9% rated the simulator as both realistic and adequate for training on each step of a cesarean section (Figure 3) [12]. 

All participants were oriented to the high-fidelity simulator prior to evaluation. Orientation included demonstrating how the simulator was assembled and discussing the simulator’s features, such as ability to replicate hemorrhage. Participants were presented a standardized scenario of a primigravid woman with arrest of dilation. They were instructed to conduct pre-operative counseling and to describe pre-operative care. Each participant was assisted during the operation by a person acting as a scrub tech who provided instruments as requested, but otherwise did not offer guidance about technique or interventions. A standard cesarean section instrument set was utilized, along with standard drapes. After delivery of the fetus, the scenario was complicated by a postpartum hemorrhage secondary to both uterine artery laceration and uterine atony. The hemorrhage scenario concluded if appropriate management occurred or after five minutes had elapsed. At this point, they were instructed to proceed with closing the uterine and abdominal incisions. When the procedure was complete, the participants were asked to verbalize postoperative orders.

**Figure 4:**
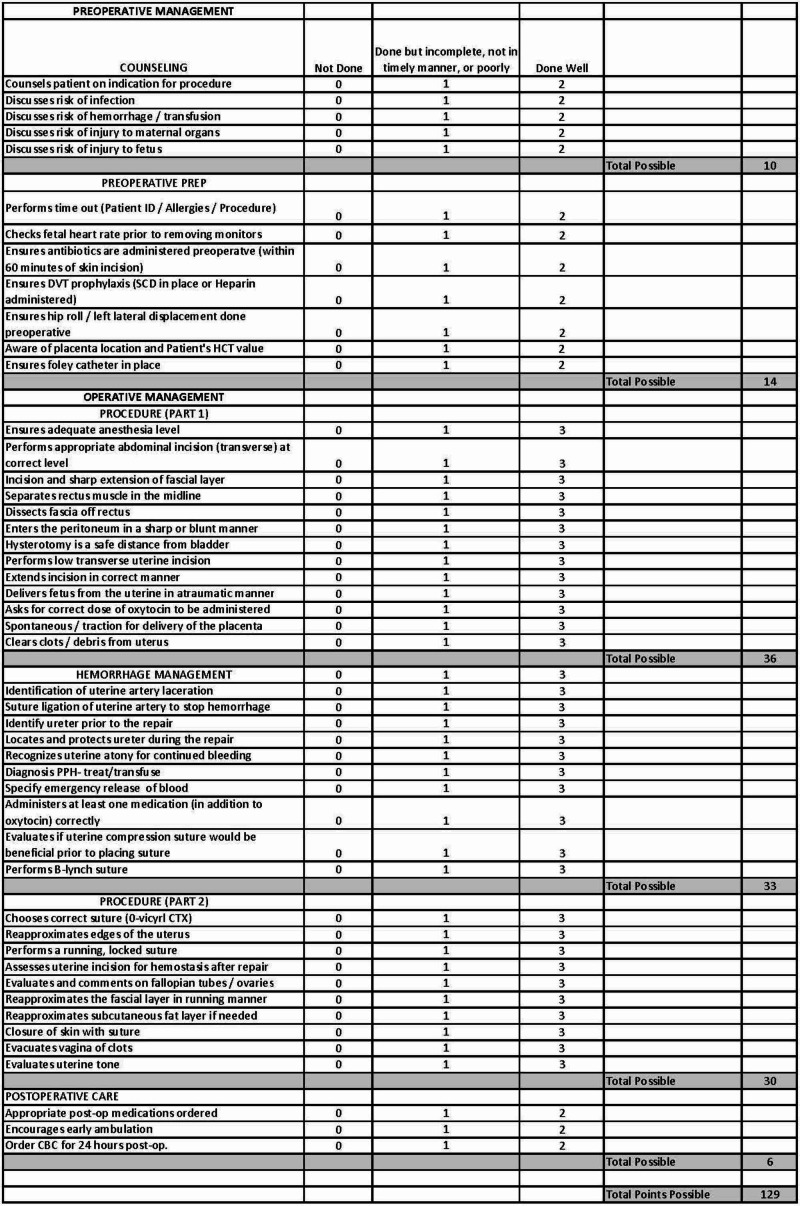
Cesarean Section Evaluation Form

**Figure 5:**
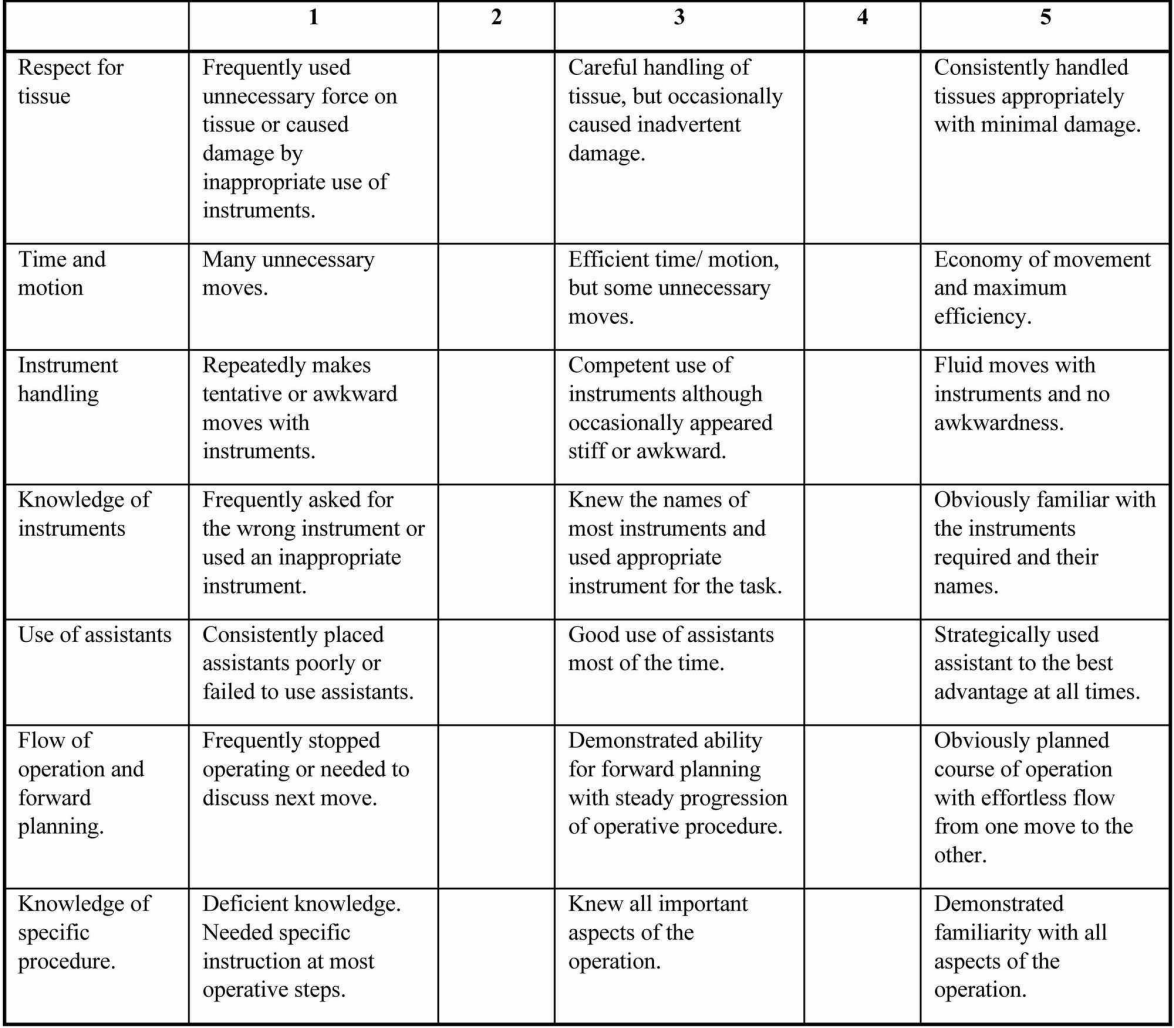
Technical Skill Evaluation (35 POINTS) Detailed global 5-point rating scale and pass/failure score for Objective Structured Assessment of Technical Skill*  [12]

**Figure 6:**
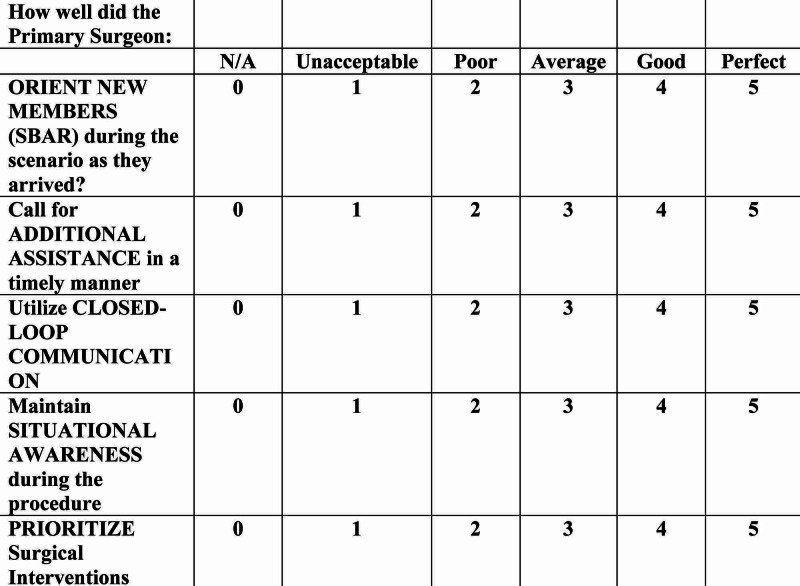
Teamwork / Communication Evaluation (25 Points)

Participants were scored using a standardized evaluation form created for each portion of the procedure (Figure 4) [13]. Additional evaluations were adapted from previously published literature to assess technical surgical skills and teamwork/communication (Figures 5, 6) [12,13]. 

Evaluators were oriented to the form and expectations of ‘done but not accurate’ versus ‘done and accurate’ by two senior investigators (LF and SD) and the same person at each facility conducted all evaluations. Participants were given a final pass/fail assessment for the entire simulation based on the entire score and the evaluators’ subjective assessment of whether or not the participant safely and effectively performed the task. 

Means and standard deviations were calculated to compare the two groups using Student’s t-test. Statistics were performed in Microsoft Excel (Microsoft Office 365). For the pass/fail assessment, proportions were compared using Fisher’s exact test.

## Results

Fifty-five eligible residents were approached for enrollment. Thirty-nine residents from three institutions consented to participate. Nineteen were randomized to the control group and 20 were randomized to the intervention group for skills training. Randomization occurred within the specialty sub-groups (OB/GYN, Family Medicine, and General Surgery). Six residents randomized to the skills task training did not attend, therefore, a total of 33 residents completed the study, 19 in the control group and 14 in the intervention group. The breakdown by specialty was 16 OB/GYN interns, 11 Family Medicine chief residents, and six General Surgery chief residents (Figure 1).

**Figure 7:**
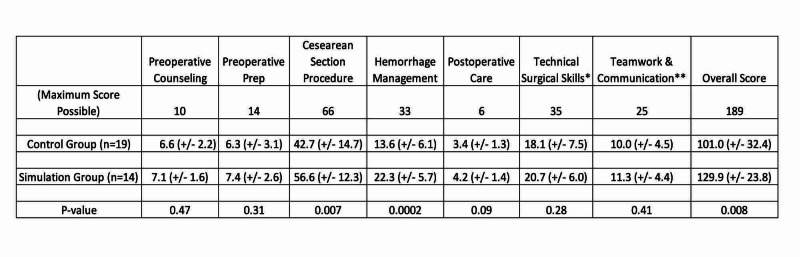
Participation Evaluation Results

The simulation trained group had significantly better performance for the cesarean section procedural steps (56.6 +/- 12.3 vs 42.7 +/- 14.7; p=0.007) as well as hemorrhage management (22.3 +/- 5.7 vs. 13.6 +/- 6.1; p=0.0002). They also had significantly higher overall scores for all aspects of the simulation (129.9 +/- 23.8) vs. 101.0 +/- 32.4; p=0.008) (Figure 7). There were no differences in preoperative counseling and management, postoperative management, overall technical skills, or teamwork (Figure 7) [12,13]. Overall, 47% of the control group passed the evaluation, while 79% of the simulation group passed, although these results did not reach statistical significance (p=0.06).

## Discussion

We found that augmenting a didactic experience with training on task simulators improved the ability to perform a cesarean delivery and manage a post-partum hemorrhage on a high-fidelity simulator. OB/GYN residents generally learn to perform cesarean sections through an apprenticeship model involving learning, reviewing, and observing the steps of the procedure, and then operating under close observation. General surgery residents do not routinely receive training in cesarean deliveries and Family Practice residents may have variable exposure depending on their residency program and interests. General Surgery and Family Practice physicians may need to perform or assist with a cesarean section in urgent cases, or if they practice in rural or low resource areas. The traditional educational construct is limited in several aspects including the unpredictability of disease occurrence and patient presentation, variations in patient anatomy, high-stress environments, and presumed increase in patient risk due to the inexperience of a novice surgeon.

Simulation allows the trainee to gain procedural experience while eliminating patient risk and decreasing cognitive stress on the learner. It facilitates the provision of safe care while still meeting the learning goals of the trainee. Simulated complications and emergencies provide the opportunity to perform multiple repetitions until comfort and proficiency are achieved. In our study, one of the most striking differences was the improved management of post-partum hemorrhage by those who underwent simulated task training.

A prior study demonstrated that incorporating a low-cost simulator plus didactics improved performance on the ability to define the steps of a cesarean section (91% versus 61.5%) and perform the procedure [5]. The results of our intervention are consistent with the finding that simulation results in an observed improvement in the performance of the cesarean section. We did not observe a difference in the ability to conduct counseling, describe pre- or post-operative management, or teamwork, which is expected as we did not include simulated counseling and team communication in our training. 

Limitations of this evaluation include the inability to evaluate procedural skills on a live patient, and lack of self-assessment of comfort with the procedure. We were also unable to control for participant use of outside educational resources or individual task practice, which may have affected performance during the testing. General surgery chief residents also have a wealth of surgical experience compared to OB/GYN interns or Family Medicine residents, and this could have influenced both their learning curve and the technical skills assessment. Due to the small sample size, we did not separately analyze data based on specialty.

The creation of a full cesarean section simulation-training program is relevant to both military and civilian providers. The majority of procedures performed for non-military female patients during humanitarian missions are for gynecologic or obstetric care. One study reported that 20% of deployed OB/GYNs performed at least one cesarean section while in a combat zone [10]. Any surgeon, OB/GYN, or Family Medicine physician who is deploying or going on a humanitarian mission may be asked to perform a cesarean delivery and manage any associated complications. There is also evidence that even obstetric residents may not feel competent in some basic tasks within their own field [9].

## Conclusions

Incorporation of a simulation-based training model into pre-deployment or pre-humanitarian mission training may improve knowledge of the procedure, confidence in performing or assisting, and enhance skills related to hemorrhage management. Further, we found that a simulation-training plan could easily be incorporated into residency programs.

Future plans include permanently incorporating cesarean section simulation into residency training, creating standardized video instruction in addition to lectures, adding simulation training for patient counseling and team communication skills, and evaluating surgical skills on live patients.
